# Causes of Kidney Graft Failure in a Cohort of Recipients With a Very Long-Time Follow-Up After Transplantation

**DOI:** 10.3389/fmed.2022.842419

**Published:** 2022-06-06

**Authors:** Michiel G. H. Betjes, Dave L. Roelen, Madelon van Agteren, Judith Kal-van Gestel

**Affiliations:** ^1^Department of Nephrology and Transplantation, Erasmus Medical Center, Rotterdam Transplantation Institute, Rotterdam, Netherlands; ^2^Department of Immunology, Leiden University Medical Center, Leiden, Netherlands

**Keywords:** kidney transplantation, ABMR, antibody-mediated rejection, TCMR, graft failure risk, long term

## Abstract

**Background:**

Biopsy-proven causes of graft loss many years after kidney transplantation are scarcely documented.

**Methods:**

Patients transplanted between 1995 and 2005 (*n* = 737) in a single center were followed on a regular basis until 2021. The recipients were divided according to age at transplantation into 3 groups; 18–39 years (young), 40–55 years (middle age), and older than 55 years (elderly). For cause biopsies of renal transplants were clustered into the categories, rejection, IFTA, return original disease, and diagnosis of *de novo* kidney disease.

**Results:**

Rejection was the main cause of graft failure censored for death at every time period after transplantation. The incidence of T cell-mediated rejection (TCMR) became rare 6 years after transplantation while the cumulative incidence of antibody-mediated rejection (ABMR) increased over time (1.1% per year). ABMR was not diagnosed anymore beyond 15 years of follow-up in recipients without pre-transplant donor-specific antibodies (DSA). An episode of TCMR was associated with an increased incidence of ABMR diagnosis in the short-term but did not increase the overall incidence of AMBR not in the long-term. Death as a cause of graft failure was an important competitive risk factor long after transplantation and resulted in a significantly lower frequency of rejection-related graft loss in the elderly group (11 vs. 23% in the young group at 15 year follow-up).

**Conclusion:**

Rejection is a major cause of graft loss but recipient’s age, time after transplantation, and the presence of DSA before transplantation determine the relative contribution to overall graft loss and the type of rejection involved.

## Introduction

Graft survival of the transplanted kidney is documented in detail for the first years after transplantation in many publications. The causes for graft loss are predominantly acute T cell-mediated rejection (TCMR), primary non-function in case of deceased donor donation, surgical complications, and increased risk of death because of cardiovascular events or infection. Data of long-term graft survival are usually derived from large registries and, in general, provide an analysis of graft loss because of death with functioning graft or graft loss censored for death. However, there is a growing interest in the causes of kidney graft loss in the (very) long term but the number of publications is still limited. A major paradigm shift has occurred by leaving the ill-defined concept of chronic allograft nephropathy (1, 2) and redefining graft loss by regularly updated pathology criteria (Banff criteria) which include the categories (chronic-active) antibody-mediated rejection (ABMR) and interstitial fibrosis with tubular atrophy (IFTA) among others (3). In particular ABMR was recognized as a major cause of kidney graft loss in the long-term (4–6). However, a close follow-up of recipients with a high degree of diagnostic biopsies was usually lacking. In addition, the role of recipients age and time after transplantation is generally not taken into account. For instance, the incidence of TCMR is recipients age-dependent and the incidence is highest within the first months after transplantation (7–10). For ABMR the relation with recipients age is not documented and whether the incidence changes in the years after transplantation is also not known.

In addition, death with functioning graft is a major cause of graft loss and is a competitive risk factor for all other causes of graft failure, particularly in the elderly. This issue is recognized but poorly addressed, although a recent publication drew attention for this cause of graft loss (11). Another recent publication, by Mayrdorfer et al., showed that the cause of graft loss changes over time after transplantation and revealed that usually a number of clinical adverse events contribute to the final progression to graft loss (12). The general lack of data of the (very) long follow-up of kidney graft recipients is likely explained by the fact that many transplantation centers do not prospectively collect their data in a dedicated database.

In this study, a cohort of kidney transplant recipients with prospective collection of relevant data and a high level of kidney biopsies-proven diagnoses was analyzed to describe the changes in cause of graft loss in different age groups over a very long time after transplantation, taking death with a functioning graft into account.

## Materials and Methods

**FIGURE 1:**
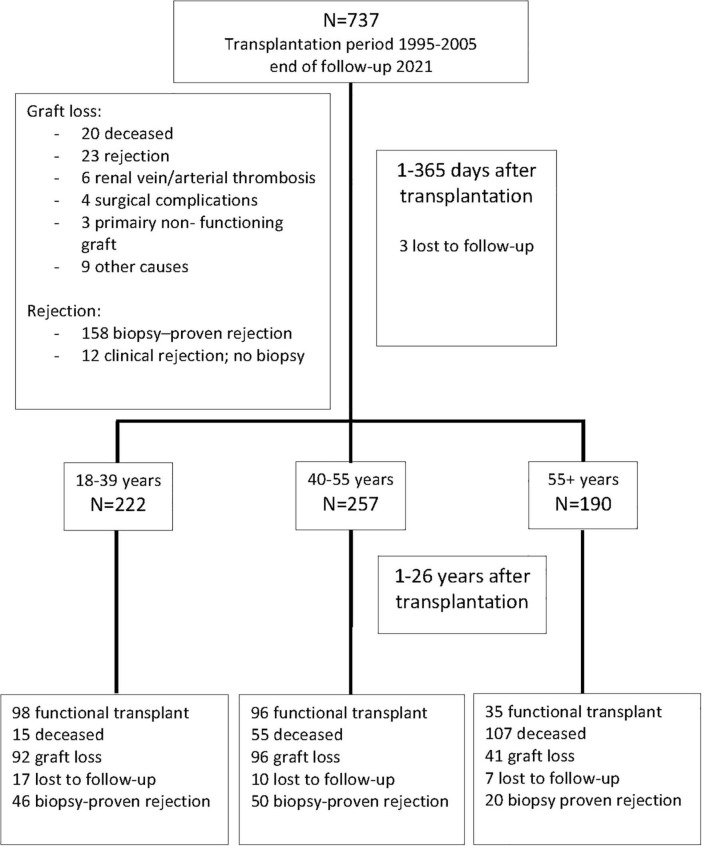
Recipients of a kidney transplant between 1995 and 2005 and causes of graft loss within the first year after transplantation. Numbers of recipients in the different age groups after 1 year are shown and the numbers lost at 25 years follow-up thereafter.

This study included all 737 kidney transplantations performed between January 1995 and December 2005 at the Erasmus Medical Center in the Netherlands. The last follow-up date before data analysis was 1 March 2021. Recipients were seen at least once a year at our out-patient clinic for follow-up and data were registered in a national database (see below) which was locally supplemented with additional clinical parameters. If the regular visits were discontinued recipients were considered lost to follow-up from their last visit. Figure 1 shows the flow chart of patients at 1 year and at the end of follow-up.

All transplantations were performed with a negative complement-dependent cytotoxicity cross-match with both current and historic sera and ABO blood group-compatible. The standard immune suppressive medication protocol with a calcineurin inhibitor was either tacrolimus (aiming for predose concentrations of 10–15 ng/ml in weeks 1–2, 8–12 ng/ml in weeks 3–4, and 5–10 ng/ml, thereafter) or ciclosporin (aiming for predose concentrations of 150–200 μg/L tapered to 100–150 μg/L at 6 months), combined with mycophenolate mofetil (starting dose of 1 g b.i.d., aiming for predose concentrations of 1.5–3.0 mg/L) and glucocorticoids. All patients received 50 mg prednisolone b.i.d. intravenously on days 0–3. Thereafter, 20 mg oral prednisolone was started and subsequently tapered to 5 mg at month 3.

For analysis, the baseline and clinical follow-up transplantation data were retrieved from the Netherlands Organ Transplant Registry (NOTR), which was over 99% complete for our center at time of this study. The clinical and research activities being reported are consistent with the Principles of the Declaration of Istanbul as outlined in the “Declaration of Istanbul on Organ Trafficking and Transplant Tourism” and in accordance with the declaration of Helsinki. The use of clinical data and assessment of donor-specific antibodies in stored serum samples was approved by the Research Ethics Committee for Biobanks and the Medical Ethics Committee of the University Medical Center Utrecht.

All renal biopsies were performed because of progressive loss of graft function and no *per* protocol biopsies were performed. All kidney biopsies were evaluated by experienced renal pathologists and commented on in detail. The original descriptions of the glomerular and tubular-interstitial compartment were used to reclassify the biopsies with rejection according to the Banff 2018 reference guide (3). Rejection episodes were classified as cellular (TCMR), humoral (ABMR), or mixed-type rejection. The latter type of rejection presented a small group (*n* = 10) and for statistical analysis these cases were combined with the humoral rejections. The presence of anti-HLA donor specific antibodies (DSA) were retrospectively measured at the pretransplant phase by Luminex single beads assay, as part of the PROCARE study (13, 14). DSA after transplantation were not routinely measured. If no serum donor-specific antibodies were present and/or C4d staining was negative or not stained for in the biopsy than the diagnosis of ABMR by histology (ABMRh, 34% of total ABMR cases) was made as described in detail before (15) and used in previous publications (16–18). The standard treatment protocol for TCMR consisted of high dose methylprednisolone (3 days of 1,000 mg per day intravenously) and T cell depletion by rabbit anti-thymocyte globulin or Alemtuzumab was added in cases of steroid-resistant rejection and/or vascular rejection. ABMR was treated with high dose methylprednisolone and intravenous immunoglobulins (1 gram/kg bodyweight) with additional plasmapheresis in early ABMR and in some selected cases Alemtuzumab as second-line treatment (15).

For data analysis the outcome of the kidney biopsy was further categorized recurrence original kidney disease (e.g., IgA nephropathy, SLE-nephritis, C3-glomerulopathy), diagnosis of *de novo* kidney disease defined as a diagnosis of primary kidney disease which was not present before transplantation (e.g., amyloidosis, post-infection glomerulonephritis) and interstitial fibrosis with tubulus atrophy (IFTA) which category contains all biopsies without a classifying diagnosis other than the presence of IFTA as a sign of chronic renal damage. Underlying kidney disease of the recipients is shown in Supplementary Table 1.

In 38 recipients, a kidney biopsy was not performed although no obvious clinical diagnosis for their progressive deterioration of graft function was present. Reviewing the charts revealed that in less than 10% this was because of refusal of the recipient, a high risk for complication or end-stage renal disease at presentation. In most cases the treating physician considered a diagnosis of “chronic allograft nephropathy” and concluded that kidney biopsy would not alter treatment policy.

If graft failure occurred the diagnosis of the *for cause* kidney biopsy was used to categorize the type of graft failure. The other outcome categories were a clinical diagnosis of cause for graft failure (e.g., acute kidney injury related to contrast/drugs-associated nephropathy, sepsis, or hemorrhagic shock) and “unknown.” The latter category contained all cases of graft failure in which no biopsy was performed and a clinical diagnosis for allograft failure could not be made.

Delayed graft function (DGF) was defined as the need for continuing dialysis after transplantation and duration of DGF was counted in days from transplantation to the last dialysis.

Within the first year after transplantation, the category “perioperative complications” was applied.

The cause of death of the recipients was documented and shown in Supplementary Table 2.

### Statistical Analysis

Three age groups were made based on age at the time of transplantation: 18–39 years (young), 40–55 years (middle age), and > 55 years old (elderly) which roughly matched to tertiles of recipients age distribution and were considered clinically relevant age categories. Differences in patient, donor, and transplant characteristics were assessed by the Fisher’s exact test for categorical variables and Mann-Whitney *U*-test for continuous variables. All *p*-values were 2-tailed.

Death censored graft loss and incidence of different causes of graft loss were assessed by Kaplan-Meier analysis with log-rank statistics for difference between strata. As all recipients had, by definition, a follow-up of 15 years, the causes of graft failure were specifically given for that point in time (Table 1). Univariate Cox proportional hazards analysis was used to identify clinical and demographic variables as given in Table 1 for their association with rejection and graft survival. Variables with a *p*-value of < 0.1 were considered for further analysis by stepwise forward regression to calculate hazard ratios and corresponding confidence intervals. PH assumption of variables were tested by visual inspection of log-minus log graphs and further tested by assessment of time-dependency using the Cox regression with time-dependent covariate module in SPSS. All variables met the demands of PH unless stated otherwise. Interaction terms that met statistical significance (*p* < 0.05) were included in the multivariate model. Normal probability plots were made and presence of significant correlations was assessed. Absence of collinearity in the model covariates was formally assessed by calculating the variance inflation factor. Statistical analysis was performed with software IBM SPSS statistics 21.

**TABLE 1:** Clinical and demographic characteristics of recipients and kidney donors given for different age categories of recipients.

	18–39 years *n* = 242	40–55 years *n* = 277	>55 year *n* = 218	*p*-value
Median age recipient in years (IQR)	30 (25–35)	47 (43–51)	61 (57–65)	
Median age donor in years (IQR)	46 (34–55)	46 (38–55)	54 (39–61)	<0.001
Recipient male/female ratio	48/52%	47/53%	45/55%	0.9
Deceased/living donor kidney	37%/63%	64%/36%	73%/27%	0.07
-DBD type*	92%	80%	81%	
-DCD type*	8%	20%	19%	0.002
-Delayed graft function	59%	50%	40%	0.3
-Duration of delayed graft function median days (IQR)	12 (6–14)	10 (9–16)	15 (9–21)	0.3
Cold ischemia time in hours	9.8 ± 0.6	11.3 ± 0.6	11.7 ± 0.7	0.3
Retransplantation	23%	19%	11%	0.003
PRA at transplantation, means (SD)	10% (21.7)	8% (18.2)	5% (16.1)	<0.001
Total HLA mismatches, means (SD)	2.2 (1.4)	2.5 (1.6)	2.9 (1.6)	0.8
Follow-up in years, median (IQR)	14.6 (5.9–19.7)	14.9 (6.6–17.7)	11.2 (6.6–18.0)	<0.001
Recipients with anti-HLA DSA at time transplantation	24.5%	21.8%	18.8%	0.4
Induction therapy	15	24	18	0.9
- Anti-IL-2 receptor antibody	13	24	18	
- T cell depleting antibody	2	0	0	
Maintenance immune suppression				0.9
- Steroids	90.4%	92.2%	92.0%	
- Tacrolimus/ciclosporin	60.3%/38.6%	59.3%/37.5%	65.9/32.0%	
- MMF/azathioprine	69.7%/0.5%	75.6%/0.0%	70.7/0.0%	
- Sirolimus	8.4%	7.5%	9.2%	
- Other	4.5%	3.1%	3.2%	

*Type of deceased donor, by brain death (DBD) or cardiac death (DCD), given as % of total deceased donor kidneys, PRA, panel reactive antibodies; DSA, donor specific antibodies; SD, standard deviation; IQR, interquartile range; P-values were calculated with Kruskal-Wallis H-test for comparing multiple independent samples.

## Results

### Baseline Characteristics and Graft and Recipient Survival per Age Category

**FIGURE 2:**
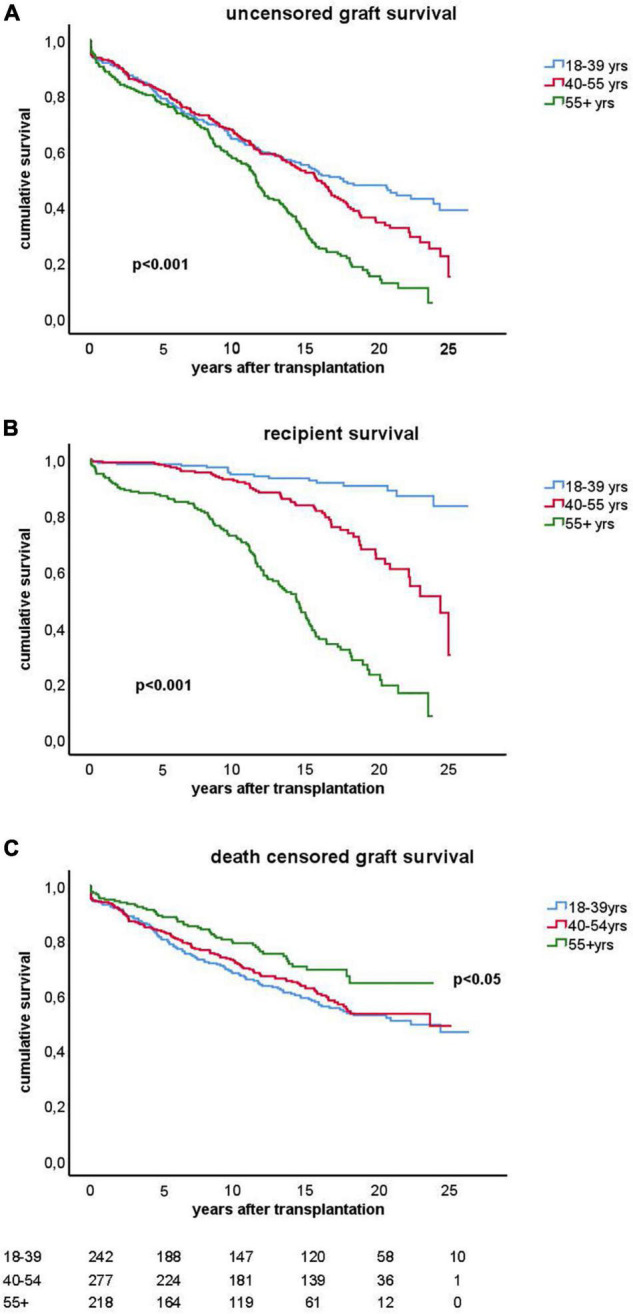
Kaplan-Meier analysis of graft survival for different age groups. The top figure **(A)** shows uncensored graft survival, the middle figure **(B)** shows the loss of grafts because of death with functioning graft and the bottom figure **(C)** shows the graft survival censored for death. *P*-values < 0.05 obtained by pooled over strata in the upper and middle figure are shown. In the lower figure **(C)**, the *p*-value for difference by log rank test statistics comparing the elderly group with the young or middle age group is given. The numbers of recipients in follow-up at different time points after transplantation are shown below the figure.

The clinical and transplant characteristics of recipients stratified according to their age category are given in Table 1. The uncensored all-cause graft survival curves for the different age groups are similar until 10 years post-transplantation (Figure 2A) after which death as cause of graft loss becomes a dominant factor (Figure 2B). Graft survival censored for death is significantly better for the elderly group as compared to the young and middle aged groups (Figure 2C) explaining the similar all-cause graft survival curves between age groups with the first 10 years after transplantation. Of note, the type of maintenance immune suppressive regimen (in particular ciclosporin or tacrolimus-based) was not associated with graft survival in the different age groups, in accordance with a previous analysis of the total PROCARE cohort (19). The majority of young recipients received a graft from a living donor as opposed to the elderly group and most of the deceased donor kidneys were from brain death donors. The incidence and duration of delayed graft function for deceased donor kidneys was similar for all age groups. Delayed graft function (and not duration) was associated with decreased graft survival (HR 1.5, CI 1.1–2.0, *p* = 0.01) but not recipient survival.

At 15 years after transplantation the frequency of death with a function graft ranges from 5.3% in the young group to 43% in the elderly group (Table 2). Within that period, rejection constitutes a major part of the known causes of graft loss censored for death in every age group; 53/89 (59%) in the young, 45/80 (56%) in the middle age, and 23/39 (58%) in the elderly group. IFTA is the second most frequent known cause of graft failure (respectively, 16, 21, and 20% in the young to elderly age category).

**TABLE 2:** Outcome at 15 years follow-up after transplantation for different categories of recipient age at time of transplantation.

	18–39 years *n* = 242	40–55 years *n* = 277	>55 year *n* = 218	*p*-value
Lost to follow-up*	14	6	9	ns
Median follow-up time at year 15 (IQR)	12.3 (6.0–15)	12.0 (6–15)	11.0 (5–15)	<0.001
Death with functioning graft	12 (5.3%)	36 (13.3%)	90 (43.1%)	<0.001
Number of graft loss other than death	95 (41.7%)	98 (36.2%)	47 (22.4%)	<0.001
Graft loss by:				
- Rejection	53 (23.2%)	45 (16.6%)	23 (11.0%)	<0.001
- IFTA	14 (6.1%)	17 (6.3%)	8 (3.8%)	ns
- Recurrence of original disease	8 (3.5%)	4 (1.5%)	1 (0.5%)	0.04
- Diagnosis *de novo* kidney disease	3 (1.3%)	2 (0.7%)	1 (0.5%)	ns
- Kidney injury/disease**	6 (2.6%)	6 (2.2%)	5 (2.4%)	ns
- Peri-operative complications	4 (1.7%)	5 (1.8%)	0 (0.0%)	ns
- Unknown	6 (2.6%)	18 (6.6%)	8 (3.8%)	ns
- Primary non-function	1 (0.4%)	1 (0.3%)	1 (0.5%)	ns

*Recipients lost to follow-up not included for calculation frequencies.

**Events or diseases causing irreversible kidney injury leading to graft loss.

*ns, not significant (p > 0.05).*

**FIGURE 3:**
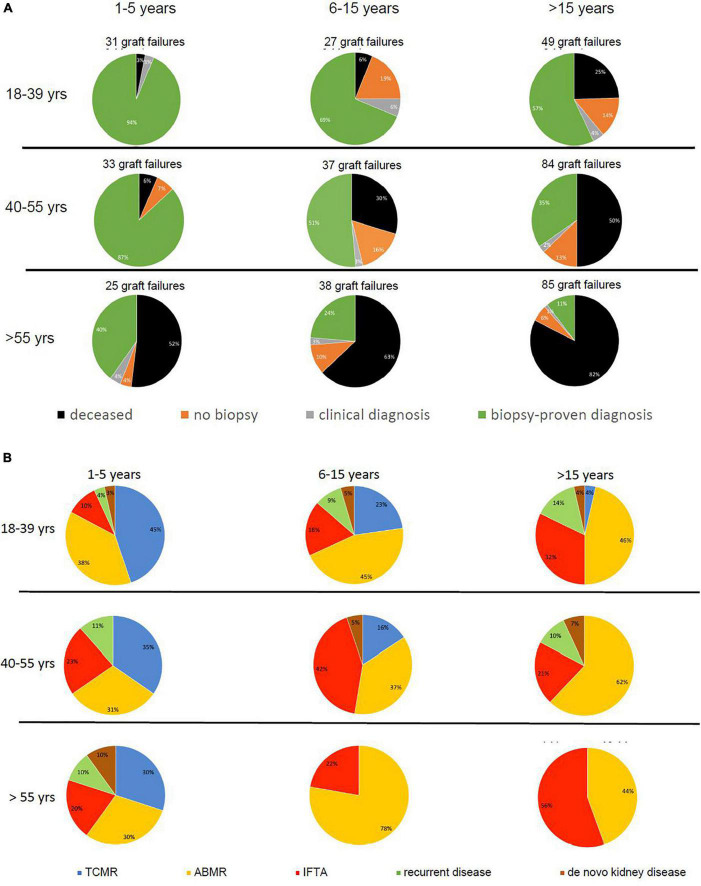
Pie charts are given for causes of graft loss in different age groups in different time periods after transplantation starting from 1 year after transplantation. In part **(A)**, the categories of cause for graft loss represent death with functioning graft, unknown (no biopsy performed and no clinical diagnosis), a clinical diagnosis of kidney injury or disease, and kidney biopsy-based cause of graft loss. In part **(B)**, the category of kidney biopsy-based cause of graft loss is split into TCMR, ABMR, return original disease, and *de novo* kidney disease. The numbers of graft loss and recipients lost at follow-up within every post-transplantation period are shown above the pie charts. Every row of pie chart represents a recipient age category at the time of transplantation (18–39, 40–55, and > 55 years) and every column represents a time period after transplantation (1–5, 5–15, and > 15 years after transplantation).

Figure 3A shows the relative contribution of causes of graft failure categorized in deceased with functioning graft, biopsy-proven, and clinical diagnosis of graft failure, and the number of “unknown causes” within in each time period after transplantation for the different age groups. Within the first year after transplantation the cause of graft failure was always identified by kidney biopsy and/or a clinical diagnosis (e.g., renal artery thrombosis, bleeding, or sepsis-related acute kidney injury) was made. Thereafter, the percentage of cases of graft failure categorized as “unknown cause” was variable per time period and age-category (Figure 3A) and on average 10% of the total number of graft losses (ranging from 0 to 19%).

Increased contribution of death to overall graft loss within the different time periods after transplantation and age categories ranged from 3 to > 80% (Figure 3A). For instance, follow-up beyond 15 years identified death with a functioning graft as a cause of graft loss in 82% in the elderly as opposed to 25% in the young group. As expected (20), malignancies, infection, and cardiovascular disease constituted the 3 main causes of death at follow-up. Relatively more infection-related death in the elderly group (17.8%) as compared to the young and middle age group (8.3% for both and *p*-value 0.08 compared to the young age group), and relatively more malignancies in the young age group (50 vs. 24.4% in the elderly age group, *p*-value 0.6) were noted (Supplementary Table 1).

### Rejection Is a Major Cause for Graft Loss but Dependent on Age and Time After Transplantation

**FIGURE 4:**
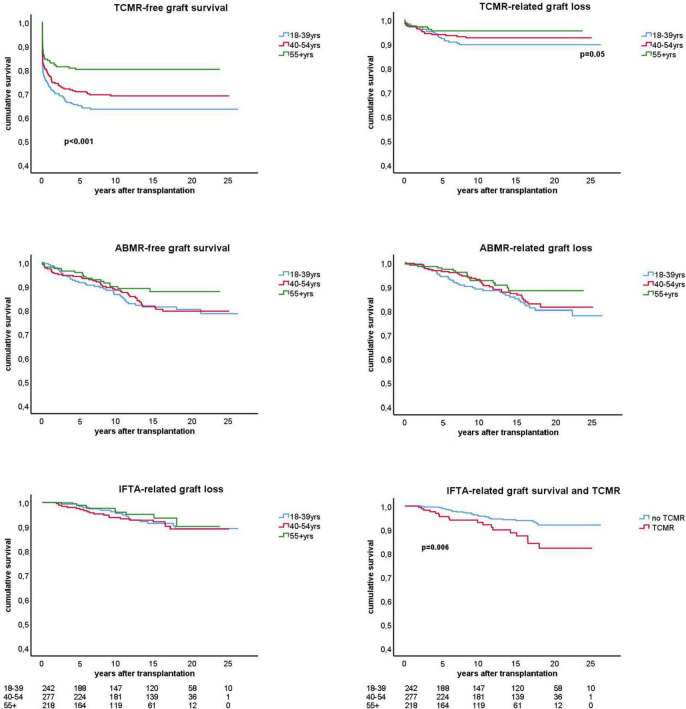
Kaplan-Meier analysis of the antibody mediated (ABMR) and T cell mediated rejection (TCMR) free-survival and the ABMR and TCMR-related graft loss for different age groups. The lower panel right shows the interstitial fibrosis and tubular atrophy (IFTA) related graft loss with a subgroup analysis for recipients with and without previous TCMR. All analysis were done by censoring for death and lost at follow-up. Number of patients in follow-up per age stratum is shown below the graphs. Only *p*-values < 0.05 are shown in the figures and obtained by log rank test statistics pooled over strata (TCMR-free graft survival), comparing the young group with the other elderly group (TCMR-related graft loss), and pairwise over strata (TCMR and IFTA-related graft loss).

In all age categories, the major cause of graft loss other than death was rejection-related (see Table 1 for 15 years follow-up and Figure 2). Figure 3B shows the relative contribution of causes of graft failure found by kidney biopsy within in each time period after transplantation for the different age groups. The risk for TCMR was clearly age and time after transplantation-related resulting in more TCMR-related graft loss in the young group (Figure 3B). After total follow-up, 23 cases in the young group had TCMR-related graft loss (9.5% of total young recipients included at time of transplantation) which was 17 cases (6.2%) and 7 cases (2.3%) in the middle age and elderly group, respectively. The incidence of TCMR became close to zero after 6–7 years for all patients. In the elderly group, graft loss because of TCMR was not observed any more after 5 years follow-up (Figures 3B, 4). The percentage of TCMR episodes leading to graft failure was on average 22.7% but age-group dependent (young: 27.4%, middle age: 20.7% and elderly recipients: 17.0%, *p* < 0.05 for trend).

**FIGURE 5:**
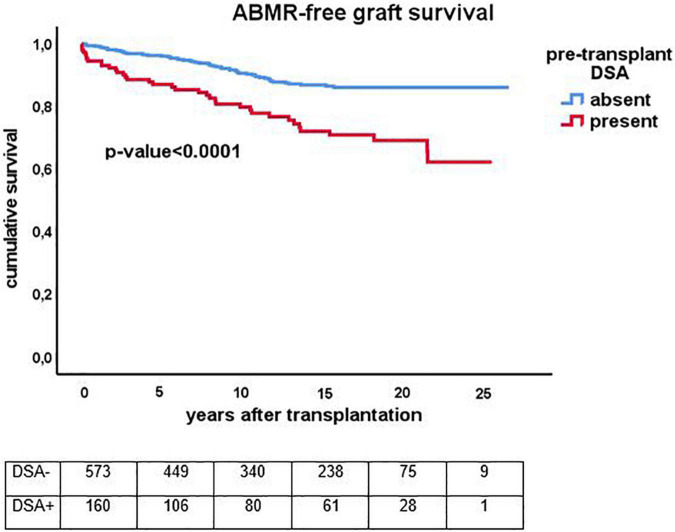
Kaplan-Meier analysis of the antibody mediated (ABMR) rejection free-survival for recipients with (*n* = 159) and without (*n* = 573) presence of pretransplant donor-specific antibodies against HLA (DSA). The *p*-value shown is obtained by comparing different strata with long rank test statistics.

The cumulative risk for AMBR increased steadily until about 15 years after transplantation after which only very few new cases were observed (Figure 4). The presence of DSA at the time of transplantation (pretransplant DSA) was a significant risk factor for ABMR and the effect persisted for many years after transplantation. New cases of ABMR diagnosed after 15 years were only observed in the recipients with pretransplant DSA (Figure 5). The average annual incidence of ABMR in the period 1–15 years after transplantation was 1.1% (range 0.7–1.5%) and unaffected by age. Uni- and multivariate logistic regression analysis (Table 3) showed several known risk factors for TCMR such as recipient’s age, cold ischemia time, positive PRA, and number of HLA mismatches. In a multivariate model, only the presence of DSA before transplantation showed a significant relation with the incidence of ABMR.

**TABLE 3:** Univariate and multivariate Cox regression analysis for risk of rejection.

	T-cell mediated rejection			Antibody mediated rejection		
	*p*-value	Hazard ratio	95% CI	*p*-value	Hazard ratio	95% CI
**Univariate analysis**						
Male sex recipient	0.99	1.00	0.751.34	0.81	1.06	0.62–1.84
Age recipient (per year)	0.001	0.98	0.97–0.99	0.13	0.99	0.97–1.04
Age donor (per year)	0.13	1.01	1.00–1.07	0.76	1.00	0.98–1.07
Deceased donor kidney	0.033	1.34	1.02–1.77	0.63	1.10	0.74–1.64
Previous transplant	0.56	1.12	0.82–1.59	0.21	1.35	0.89–2.20
Number of HLA mismatches	0.001	1.16	1.06–1.26	<0.001	1.26	1.11–1.43
PRA positive (>5%)	0.003	1.55	1.16–2.06	0.08	1.45	0.96–2.21
Cold ischemia time per hour	0.002	1.02	1.01–1.03	0.98	1.00	0.98–1.01
Pretransplant DSA present	0.38	1.15	0.83–1.59	<0.001	2.18	1.43–3.32
**Multivariate analysis**						
Age recipient (per year)	<0.001	0.96	0.97–0.99	−	−	−
Cold ischemia time per hour	<0.001	1.03	1.01–1.04	−	−	−
Pretransplant DSA present	−	−	−	<0.001	2.24	1.46–3.43
Number of HLA mismatches	<0.001	1.25	1.14–1.38	−	−	−
PRA positive (>5%)	0.02	1.41	1.06–1.89	−	−	−

The percentage of biopsy-proven ABMR cases leading to graft loss at the end of follow-up was high (74.4%) and tended to be higher in the young recipient group (young: 83.7%, middle age: 69.0% and elderly recipients:68.4%, *p* = 0.2). The much higher risk for graft loss because of death in the elderly group obviously greatly reduced the impact of ABMR on graft survival. For instance, in the period 15–26 years after transplantation the relative and absolute number of cases with ABMR-related graft failure in the elderly group was significantly lower (4 cases; 5% of total graft loss) as compared to the younger group (13 cases; 27% of total graft loss, *p* < 0.01, Figure 3B). In other words, although the risk for ABMR-related graft loss is similar for all age categories, only 14 recipients in the elderly group (6.4%) had lost their graft because of ABMR after 1–26 years, compared to 15.3 and 12.8% in in the young and middle age group (*p* = 0.01).

**FIGURE 6:**
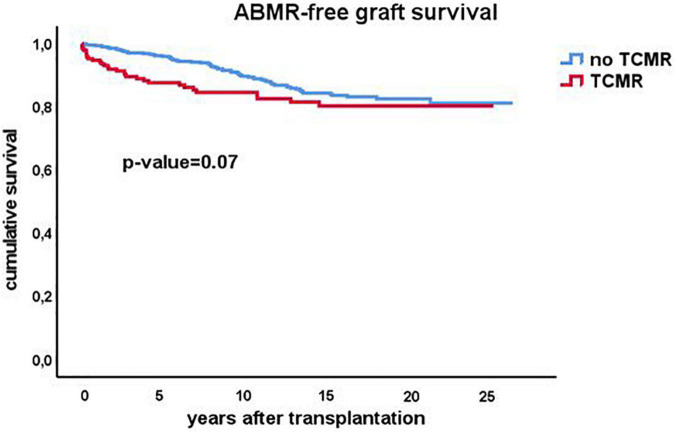
Kaplan-Meier analysis of the antibody mediated (ABMR) rejection free-survival for recipients with (*n* = 207) and without (*n* = 530) a previous episode of TCMR. The *p*-value shown is obtained by comparing different strata with long rank test statistics.

As early TCMR may be a risk factor for later development of ABMR, the ABMR-free survival Kaplan-Meier curves were made for recipients with and without an episode of TCMR after transplantation. Interestingly, TCMR was associated with an increased incidence of ABMR diagnosed earlier after transplantation but survival lines converged after 10 years with overall no difference in the cumulative incidence of ABMR (Figure 6).

### Interstitial Fibrosis and Tubular Atrophy-Related Graft Loss Is Independent of Recipients Age and Influenced by Previous T Cell-Mediated Rejection

The risk for graft loss with a biopsy-proven diagnosis of chronic damage was independent of age (Figure 4) and it became a relatively more frequent cause of graft loss long after transplantation (Table 1 and Figure 3B). At 15 years follow up, the percentage of graft loss because of IFTA was 5.5% (Table 1). Beyond 15 years of follow up, ABMR and IFTA were the dominant, almost exclusive, causes of graft loss (Figure 3B).

A previous rejection may lead to IFTA and subsequent graft loss at longer follow-up (2). To test this hypothesis we made separate KM curves for IFTA-related graft loss for recipients with and without rejection. Only TCMR was significantly related to IFTA-related graft loss (Figure 4, bottom right figure) which was confirmed by logistic regression analysis (HR 2.3, *p* = 0.008). At maximal follow-up, 27 out of 369 recipients (7.3%) with no TCMR episode (60% of all IFTA-related graft loss) and 18 out of 93 recipients (19.3%) with a previous TCMR had IFTA-related graft loss (*p* = 0.001).

### Recurrence of Original Disease

Graft failure because of recurrent disease was relatively rare with 15 identified cases (2.0% of total recipients) with great diversity in biopsy diagnosis; IgA nephropathy (*n* = 2), auto-immune vasculitis (*n* = 3), diabetic nephropathy (*n* = 3), membranoproliferative glomerulonephritis (*n* = 3), thrombotic microangiopathy (*n* = 1), and focal segmental glomerulosclerose (*n* = 3). As expected based on the higher frequency of glomerulonephritis and glomerulopathy (Supplementary Table 1), recurrence of the original disease was predominantly noted in the young (*n* = 8, 3.3%) and middle age groups (*n* = 6, 2.2%) with only 1 case in the elderly group (0.5%) which is illustrated by Figure 3B.

### Diagnosis of *de novo* Kidney Disease

*De novo* kidney disease was rarely encountered as a cause for graft failure and documented in 8 recipients (1.0% of total recipients); BKV nephropathy (*n* = 2), JC-virus nephropathy (*n* = 1), tubulo-interstitial nephritis (*n* = 1), cholesterol emboli (*n* = 1), diabetic nephropathy (*n* = 2), and anti-GBM disease in a recipient with Alport disease (*n* = 1).

## Discussion

This analysis of a very long-term follow-up study of kidney transplant recipients up to 26 years is first in its kind to show that causes of graft failure are a function of post-transplantation time and recipient’s age. The data obtained in this study indicate that TCMR is in particular contributing to graft loss in the young patients but the impact becomes negligible after 5 years post-transplantation and about 2 years earlier in the elderly recipients. The incidence of AMBR in for cause biopsies is remarkably constant in the period of 1–15 years after transplantation and not age-dependent.

After 15 years, there are very few new cases of ABMR and similar to TCMR, the ABMR-free survival curve flattens. A previous TCMR increases the incidence of ABMR shortly after transplantation but in the long run there is no influence of TCMR on the cumulative incidence of ABMR. Taken together the data suggest that a particular load of antigenic mismatches is required to develop ABMR, in accordance with recent studies on the association between the number of predicted indirect recognizable donor-derived HLA epitopes (PIRCHE) which can be presented by recipients HLA class II and long-term graft survival (21, 22). The cumulative incidence of ABMR is probably dependent on the intensity of the immune suppressive drug regimen and there are no data to support the hypothesis that after 15 years tolerance is achieved. However, the current data do imply that at least long after transplantation not only the risk of TCMR but also the risk of ABMR becomes very low. The latter seems to apply in particular to the group of recipients without the presence of DSA before transplantation. The data from this study suggests that pretransplant DSA cause an increase in the risk for ABMR which persists even many years after transplantation. The data from this cohort study emphasizes the important role of the anti-donor humoral response in (long-term) graft loss as was already postulated almost 20 years ago by Terasaki (23).

For clinical decision making, it is important to realize that death with functioning graft is a major competitive risk for long term causes of graft loss, in particular ABMR. Elderly recipients with a high burden of comorbidities will have a limited life span after transplantation although they may still benefit from kidney transplantation over continuing dialysis (24, 25). The mortality of recipients in the long term has improved over the decades but is still substantial in the elderly (20). Therefore, the *a priori* chance of losing the kidney graft because of ABMR in these vulnerable recipients is relatively small. In contrast, young patients have a substantial risk for graft loss because of either TCMR in the first couple of years and ABMR, thereafter. These recipients will have the greatest benefit of a well HLA-matched kidney allograft.

Recurrence of original kidney disease, newly diagnosed kidney disease, and clinically diagnosed causes of graft failure beyond 1 year post-transplantation are relatively infrequent causes of allograft failure.

The strength of our study is the unique long and close follow-up of the recipients with relative few lost to follow-up and a high score of *for-cause* kidney biopsies. However, we realize that such a prolonged observation period as in this study introduces many confounders which are difficult to account for. For instance, immune suppressive drug regimens have changed over time and within patients. In addition, it is unclear to what extent the result of our center can be generalized, in particular, with respect to the incidence of death with function graft. In our center we tend to have a relative liberal transplantation policy with respect to the eligibility of patients with a high co -morbidity score (24, 26). However, the overall graft survival for deceased donors at 5 (72%) and 10 years (58%) observed in this study is very similar to the European data from the ERA-EDTA and the CTS registry obtained in the same era of transplantation (27). Moreover, death with functioning graft in the different age groups at different time intervals after transplantation is similar to the aforementioned CTS registry data.

In accordance with other studies, delayed graft function in the recipients receiving a deceased donor kidney negatively impacts the graft survival (28) and underlines the need for preventing this adverse event (29). Not only may it lead to delayed graft function to primary non-function of the kidney but it also impacts the long-term survival of kidneys which may be mediated by a significantly lower eGFR at 1-year post-transplantation (30).

Although we recognize that graft loss may have many contributing factors as recently demonstrated (12), this usually concerns renal hits that cause a transient or permanent decrease but not progressive loss of eGFR. Only when, for instance, pneumosepsis led to irreversible loss of graft function, with return to dialysis, this was registered as the cause for graft failure in our study. In all other cases, the kidney biopsy was performed because of a steadily declining graft function. The relative contribution of the category “unknown” was, on average, relatively small although quite variable per age group and time period. The impact on the overall results was judged as marginal, in particular, as no specific bias could be identified for not performing a diagnostic renal biopsy.

In summary, this study shows the impact of rejection on graft failure as a function of time-after-transplantation and age with death as a very strong competitive risk factor in the elderly recipients. For the younger recipients, ABMR in the long term is a dominant cause of graft failure, specifically in the group with pre-transplantation DSA. A plateau in the cumulative incidence of ABMR in the group without pretransplant DSA suggest that particular or total number of epitope mismatches are important determinants for the absolute risk for ABMR-related graft loss over a prolonged time of follow up.

## Data Availability Statement

The original contributions presented in this study are included in the article/Supplementary Material, further inquiries can be directed to the corresponding author.

## Ethics Statement

The studies involving human participants were reviewed and approved by the Research Ethics Committee for Biobanks and the Medical Ethics Committee of the University Medical Center Utrecht. The patients/participants provided their written informed consent to participate in this study.

## Author Contributions

MB participated in research design, writing of the manuscript, and data analysis. DR participated in research design and writing of the manuscript and provided analytical tool. MA and JK participated in data analysis and writing of the manuscript. All authors contributed to manuscript revision, read, and approved the submitted version.

## Conflict of Interest

The authors declare that the research was conducted in the absence of any commercial or financial relationships that could be construed as a potential conflict of interest.

## Publisher’s Note

All claims expressed in this article are solely those of the authors and do not necessarily represent those of their affiliated organizations, or those of the publisher, the editors and the reviewers. Any product that may be evaluated in this article, or claim that may be made by its manufacturer, is not guaranteed or endorsed by the publisher.

## Abbreviations

ABMR: antibody-mediated rejection
CDC: complement dependent cytotoxicity
C4d: complement C4d
DSA: donor-specific antibodies
IFTA: interstitial fibrosis and tubular atrophy
PRA: panel reactive antibodies
TCMR: T cell-mediated rejection.
