# Supplementary material for: Causes of Kidney Graft Failure in a Cohort of Recipients With a Very Long-Time Follow-Up After Transplantation
Source: Front Med (Lausanne). 2022 Jun 6;9:842419. doi: 10.3389/fmed.2022.842419 (PMC9207199; doi:10.3389/fmed.2022.842419)
Supplement: Supplementary file 1 [file Table_1.pdf]

Table S1. Underlying kidney disease of kidney transplant recipients per age category

	18-39 years	40-55 years	>55 years	All recipients
Hypertensive nephropathy/atherosclerosis	13,6%	22,4%	46,4%	30,3%
Glomerulonephritis//glomerulopathy	33,3%	28,2%	15,5%	24,1%
- IgA nephropathy	10.6%	8.2%	2.7%	
- FSGS	3.0 %	4.7%	2.7%	
- MPGN	1.5%	3.5%	0.9%	
- SLE/vasculitis	6.0%	8.2%	3.6%	
- Other	12.1%	3.5%	5.4%	
Diabetic nephropathy	7,6%	10,6%	10,9%	10,0%
Nephrolithiasis	0,0%	1,2%	0,9%	0,8%
Reflux nephropathy	19,7%	4,7%	0,0%	6,5%
Polycystic disease	4,5%	17,6%	10,9%	11,5%
Other	15,2%	7,1%	8,2%	9,6%
Unknown	6,1%	8,2%	7,3%	7,3%
Count	66	85	110	261
