# Supplementary material for: Causes of Kidney Graft Failure in a Cohort of Recipients With a Very Long-Time Follow-Up After Transplantation
Source: Front Med (Lausanne). 2022 Jun 6;9:842419. doi: 10.3389/fmed.2022.842419 (PMC9207199; doi:10.3389/fmed.2022.842419)
Supplement: Supplementary file 2 [file Table_2.pdf]

Table S2 Causes of death of recipients with a functioning kidney allograft after 15 year follow up stratified for different age categories at time of transplantation

	18-39 yrs	40-54 yrs	>55 yrs	All recipients
Number of deceased recipients at follow-up	12	36	90	138
Causes of death				
- Malignancy	6 (50%)	14 (38,9%)	22 (24,4%)	42 (30,4%)
- Infection	1 (8,3%)	3 (8,3%)	16 (17,8%)	20 (14,5%)
- Cardiovascular disease	3 (25,0%)	5 (13,9%)	22 (24,4%)	30 (21,7%)
- Other	1 (8,3%)	5 (13,9%)	15 (16,7%)	21 (15,2%)
- Unknown	1 (8,3%)	9 (25,0%)	15 (16,7%)	25 (18,1%)
